# Feed Supplementation with a Commercially Available Probiotic Solution Does Not Alter the Composition of the Microbiome in the Biofilters of Recirculating Aquaculture Systems

**DOI:** 10.3390/pathogens9100830

**Published:** 2020-10-10

**Authors:** Simon Menanteau-Ledouble, Rui A. Gonçalves, Mansour El-Matbouli

**Affiliations:** 1 Clinical Division of Fish Medicine, Department for Farm Animals and Veterinary Public Health, University of Veterinary Medicine, Veterinärplatz 1, A-1210 Vienna, Austria; Mansour.El-Matbouli@vetmeduni.ac.at; 2 Department of Chemistry and Bioscience, Aalborg University, Frederik Bajers Vej 7H, DK-9220 Aalborg, Denmark; 3 Lucta S.A., Innovation Division, Research Park, Edifici Eureka—MRB, Campus UAB, Bellaterra (Cerdanyola del Vallès), 08193 Barcelona, Spain; ruialexandre.goncalves@lucta.com

**Keywords:** biofilms, nitrifying bacteria, *Planctomycetes* sp., *Chloroflexi* sp., *Rhizobium* sp.

## Abstract

Recirculating aquaculture relies on the treatment of ammonia compounds from the water by a bacterial flora growing inside biofilters. Another increasingly common practice in aquaculture is the supplementation of feed with live probiotic bacteria to boost the immune system of the farmed animals and hinder the implantation of pathogenic bacteria. In the present study, we investigated the bacterial flora within the biofilters of recirculating farming units in which African catfish (*Clarias gariepinus*) were being farmed. Our results suggested that these two farming systems could be compatible as feeding of the probiotic feed had no detectable effect on the composition of the microbiome within the biofilters and none of the bacteria from the feed could be detected in the biofilters. These findings suggest that supplementation of the fish feed with probiotic supplements did not interfere with the microbiome residing inside the biofilter and that it is a safe practice in recirculating aquaculture systems.

## 1. Introduction

Aquaculture has been the fastest growing animal food-producing sector for several decades and production from aquaculture is expected to almost double in the next decade [1,2]. However, the industry is still hindered by the occurrence of infectious diseases [3,4], especially considering that outbreaks of bacterial diseases are still largely treated through the application of antibiotics, which has become an urgent public health issue in the face of rising antibiotic resistance [5]. Both the application of probiotics and cultivation in recirculating aquaculture systems (RAS) have been growing trends for the aquaculture industry over the last decades. Probiotics are live bacterial preparations with a beneficial effect on the biology of the animal. For example, providing enzymes assisting with the host biological processes, including immunostimulation [6]. Other probiotics contain bacteria that can hinder potential pathogens by competitive exclusion or antimicrobial activity against potential pathogens [7,8]. Likewise, some probiotics have been shown to interfere with the virulence mechanisms of bacteria—for example, by inactivating molecules involved in the quorum sensing apparatus, reducing the expression of virulence factors by the pathogens [9,10]. Notably, a common source of probiotic bacteria, including in aquaculture settings, is lactic acid-producing bacteria, commonly from the *Bacillus* genus. *Bacillus* bacteria have demonstrated direct antimicrobial [6] and quorum-quenching abilities [10].

RAS are farming or cultivation systems in which a large portion of the effluent water is treated and supplied back to the fish, very strongly reducing the need for the input of outside water [11,12]. A critical step in this treatment of water is the removal of self-polluting compounds excreted by the fish, in particular nitrogenous compounds such as ammonium and nitrates that are known to be highly toxic to fish. These compounds are converted through a two-step process in which ammonia is first oxidized into nitrite, followed by a nitrification step where nitrites are converted into nitrates and dinitrogen by nitrifying bacteria hosted in a biofilter unit [13,14]. In practice, upon completion, the RAS is run for several weeks until nitrifying bacteria can colonize the filter. These nitrifying bacteria are often seeded to the biofilter, for example by including biofilter media from a previously established biofilter, although placing a small number of fish inside the system has also been performed [15]. This method can be dangerous for the animals as the filter is not yet ready to remove nitrogenous compounds and their levels will rise rapidly in the RAS. RAS generally include a step of antimicrobial disinfection to prevent the accumulation of bacteria in the water; this is often performed by UV disinfection because of its relative low-cost and safety to the fish, although ozone is also routinely used instead, although it is considered more dangerous for the fish and biofilters [16]. In addition, alternative methods of sterilization are occasionally used but these are comparatively rare. The placement of this sterilization unit within RAS varies between systems, with it often being placed either immediately before or after the biofilter unit.

Because biofilters rely on a population of bacteria to perform this removal of nitrogenous compounds, there is a concern that the systemic addition of live bacteria to the tank, especially bacteria with known antimicrobial properties, could displace or otherwise interfere with the native bacteria in the biofilter. Because of the central role of the biofilter units in RAS, it is therefore important to confirm that probiotic supplementation does not interfere with their native microbiome or function.

## 2. Materials and Methods

### 2.1. Experimental Units, Fish, and Feed

Four recirculating units were used. These units were set up in parallel and each was independent and had its own biofilter. The turnover time in the units was approximately 45 min to one hour and new water, totaling about 10 L per week in average, corresponding to a daily volume of make-up water slightly below 1% of the total volume, was added once a week to compensate for losses due to splashes and evaporation. Water temperature was maintained at 28 ± 1 °C and water quality parameters, notably concentration of nitrate and nitrites, were monitored daily throughout the experiment using commercially available water quality strips (EasyTest, JBL, Neuhofen, Germany) and sterilization was performed using UV disinfection lamps (5.0 mJ/cm^2^) located immediately downstream from the biofilters. At the time of the start of the experiment, the water system had been running continuously for two months and the biofilters were considered mature, as confirmed by measuring the biofilter’s ability to degrade nitrogenous compounds.

Each RAS was organized around a tank (1 m^3^) that contained 20 African catfish (*Clarias gariepinus*), previously acquired from a local importer (Fisch Farm Hochwimmer & Partner, Wirtschaftspark II/7 7032 Sigless, Austria), held at a density of 15 kg.m^−3^ and fed using a commercial pelleted diet (Skretting) at 3% body weight per day. In two of these RAS, this diet was supplemented with a commercial probiotic preparation (Biomin Aquastar Growout, Biomin, Getzersdorf, Austria) added to the feed according to the manufacturer’s instructions (3.5 g/kg of feed), resulting in a duplicate. Aquastar Growout is a commercially available probiotic solution composed of a mixture of four bacterial species (*Enterococcus* sp., *Pediococcus* sp., *Lactobacillus* sp., and *Bacillus* sp.). Members of these various genera all have well-established potential as probiotics in fish and the Aquastar formula was previously found to be associated with increased farming performances in tilapia (*Oreochromis niloticus*) [17] as well as whiteleg shrimp (*Litopenaeus vannamei*) [18]. This mixture was also found to have immunostimulatory properties in *O. niloticus* [19] and to delay mortalities and improve survival in populations of *L. vannamei* following both natural and artificial infections with *Vibrio parahaemolyticus* [18,20].

### 2.2. Sample Collection

Moving bed biofilm reactor (MBBR) materials, 50 mm in diameter, were collected from each of the biofilter units at the start of the supplemented feeding (25th of February) and approximately three months later (30th of May). The biofilters were sampled twice—at the beginning of the experiment and then three months later. Layers of biological material (biofilms) attached to the biomedia were scraped off from these MBBRs using a scalpel blade and placed into Eppendorf tubes in technical triplicates, and after collection of the biofilms, genomic DNA was extracted using a DNeasy kit (Qiagen, Hilden, Germany) according to the manufacturer’s instructions. The extracted genomic DNA preparations were analyzed using a nanodrop (Thermo Scientific, Waltham, Massachusetts, United States) to confirm the concentration and quality of the DNA. The DNA was then diluted to an identical concentration of 100 ng/L. Then, 16S rRNA sequences were amplified using the degenerate universal primers Bakt_341F (CCTACGGGNGGCWGCAG) and Bakt_805R (GACTACHVGGGTATCTAAKCC) [21]. Afterwards, 300 bp paired-end read sequencing of the resulting amplicons was performed (LGC genomics Germany, Berlin, Germany) using an Illumina MiSeq V3 (Illumina, San Diego, California, United States), resulting in a total of one million read pairs. Demultiplexing of all libraries for each sequencing lane was performed using the Illumina bcl2fastq 1.8.4 software. Clipping was performed to remove primer sequences and sequencing reads with a final length inferior to 100 bases in length.

Identification of the various bacterial species in the biofilter samples was performed manually at the genus or, when possible, species level, based on the sequences of these amplicons based on homology with the database Genbank (National Center for Biotechnology Information, Bethesda, MD, USA). The total read count for each amplicon was further used to estimate the relative prevalence of each bacterial genus and these were compared between treatments (probiotic diet compared to unsupplemented diet) and time points.

Read counts for the amplicons were analyzed using IBM SPSS Statistics 25 (Microsoft, Redmond, WA, USA); only the amplicons with read counts over 1000 were kept and the others were grouped into the same “Other” groups. A Levene test was performed to determine if these counts were normally distributed. As a normal distribution was found for these read counts, an analysis of variance (ANOVA) was performed according to the treatment of the RAS (probiotic-supplemented feed or not) and the time of sampling. Then, because the read counts according to the treatment (control vs. probiotics) were not normally distributed, a non-parametric Kruskal–Wallis test was performed instead.

## 3. Results and Discussion

A large number of different bacteria were identified in all samples, suggesting that the microbiome in the filters was diverse rather than dominated by a small numbers of bacterial species. Similarly, several bacteria were either not or poorly identified, sometimes only at the phylum level. Indeed, multiple amplicons were identified as “uncultured bacteria”, and on occasion, several species of bacteria matched the amplicon equally well. This probably represents the biggest limitation of the present results and is likely an illustration of the relative scarcity of our knowledge regarding environmental bacteria. Understandably, most of the history of microbiology has been dominated by the study of bacterial pathogens or bacteria otherwise of economic importance. Conversely, despite constituting the majority of bacteria, environmental bacteria have received much less scrutiny.

As expected, the dominant species of bacteria identified in every samples were environmental and nitrifying bacteria, in particular belonging to the *Planctomycetes*, *Chloroflexi* and *Rhizobium* genera [22,23,24]. The most common inhabitants of the biofilter were members of the genus *Chloroflexi*, closely followed by *Planctomycetes* sp. and *Chitinophaga* sp. in the third place (Table 1). Neither *Planctomycetes* sp. nor *Chloroflexi* are considered to be nitrifying bacteria; however, they are frequently reported from biofilters. Indeed, our results are, overall, somewhat similar to the results reported by Hüpeden et al., who surveyed microbial communities in different fresh, brackish and saltwater recirculating systems and reported that *Planctomycetes* sp. and *Chloroflexi* sp. were among the most common bacteria, alongside *Proteobacteria* sp., *Bacteriodetes* sp. and *Nitrospirae* sp [25]. Likewise, *Planctomycetes* sp., *Chloroflexi* sp., *Chitinophaga* sp. and *Rhizobiaceae* were among the bacteria reported by Brailo et al. following their screening of a pilot seawater biofilter unit [26]. In contrast, Bartelme et al. have reported that *Nitrosomonas* sp., *Nitrospira* sp. and *Nitrobacter* sp. were the bacteria most consistently present in their biofilter samples originating from a commercial scale freshwater RAS [27]. These bacteria are known to play an important role in the nitrification two-step process, and while these genera of bacteria were also found in all of our samples, it was always at a relatively low number compared to many other bacterial genera.

The specific representation of the various genera varied between samples, including between replicates within the same treatment and between sampling points, including in the same biofilter. This is consistent with the findings reported by Bartelme et al. who indicated that the precise composition of the microflora in their biofilter varied overtime, albeit while conserving a stable core composed of a mixed population of *Archaea* and *Nitrospira* bacteria [27]. However, these changes over time were limited in our experiment and ANOVA did not confirm any significant effect of the time of sampling on the distribution of the amplicons (*P* = 0.558).

More importantly, there was no difference between the composition of the microbiomes in the RAS that had received the commercial feed and the ones that had received the feed supplemented with the probiotic solution, as analyzed using a non-parametric Kruskal–Wallis test (*P* = 0.772). These results are consistent with the fact that none of the bacteria that composed the probiotics feed could be identified in the samples. Moreover, the water parameters were monitored daily throughout the trial and were found to remain well within safety margins at all time. In particular, nitrogenous compounds, NO_3_ and NO_2_, when detectable at all, remained at the lowest limit of detection (1 mg/L and 0.5 mg/L, respectively). This is consistent with the idea that the biofilter’s function was not impaired during the experiment.

In the present study, administration of probiotics was not found to have a detectable effect on the composition of the biofilter microbiome and did not appear to lead to the displacement of the bacteria already existing in the biofilters. Administration of probiotics aims at altering the composition of the fish intestinal microflora [28]. However, results from the present study suggest that these changes did not transfer to the biofilter units. A likely explanation is that these probiotic bacteria were less adapted and out-competed by the already implanted populations of nitrifying bacteria.

While these results are interesting, it remains unclear how applicable they would be to other combinations of RAS and probiotics; in the future, it would be to other combinations of RAS and probiotics. Moreover, it would be of interest to repeat this experiment with a higher number of treatments for each replicate. There can be a high level of variability between each biofilter and so the effect of a treatment is difficult to detect. In the present experiment, we were limited by practical considerations, in particular the number of available RAS, but it would be beneficial to repeat this experiment on a larger scale with more units. Similarly, the experiment was only performed for a duration of three months. It would be of interest to see if the effect of the probiotic might get stronger over time. Finally, we elected to use a commercial solution of probiotics that was already available to producers and is known to be adapted for fish species [17,19]. In future experiments, it might be of interest to mark the bacteria’s genome with unique genetic markers to make their detection in the biofilter easier.

In conclusion, we did not find any significant alteration in the biofilters’ microbiome following feeding with the probiotic-supplemented feed. Therefore, our results suggest that supplementation of fish feed with probiotics might have only a limited effect on the biofilter microflora, which would make them safe for use on RAS, although more research is needed to confirm these results. Probiotics are beneficial in many ways and their application in RAS would make these companies both more productive and more environmentally friendly [7,8].

## Figures and Tables

**Table 1:** The five most common bacteria for each samples at the time of the second sampling.

Control A		Control B		Probiotics A		Probiotics B	
Most Common Bacteria	Read Counts	Most Common Bacteria	Read Counts	Most Common Bacteria	Read Counts	Most Common Bacteria	Read Counts
*Planctomycetes* sp.	12,906	*Chloroflexi* sp.	6142	*Chloroflexi* sp.	21,679	*Chloroflexi* sp.	16,001
*Chitinophaga* sp.	9586	*Planctomycetes* sp.	5041	*Planctomycetes* sp.	11,622	*Planctomycetes* sp.	12,180
*Ramlibacter* sp.	9030	*Bradyrhizobium* sp.	2845	*Microbacterium paraoxydans*	2810	No match	3596
*Acidovorax* sp.	8561	*Chitinophaga* sp.	2710	*Chitinophaga* sp.	2645	*Microbacterium paraoxydans*	3572
No match	5346	*Mesorhizobium* sp.	2447	*Mesorhizobium* sp.	2550	*Mesorhizobium* sp.	3517

## Data Availability

The datasets used and/or analyzed during the current study are available from the corresponding author on reasonable request.

## References

[1] Food and Agriculture Organization of the United Nations (FAO) (2018). The State of World Fisheries and Aquaculture Meeting the Sustainable Development Goals.

[2] The World Bank (2013). FISH TO 2030: Prospects for Fisheries and Aquaculture.

[3] Leung T.L.F., Bates A.E. (2013). More rapid and severe disease outbreaks for aquaculture at the tropics: Implications for food security. J. Appl. Ecol..

[4] Adams A., Thompson K.D. (2011). Development of diagnostics for aquaculture: Challenges and opportunities. Aquac. Res..

[5] O’Neil J. (2016). Tackling Drug-Resistant Infections Globally: Final report and recommendations. Review on Antimicrobial Resistance.

[6] Urdaci M.C., Bressollier P., Pinchuk I. (2004). *Bacillus clausii* Probiotic Strains: Antimicrobial and Immunomodulatory Activities. J. Clin. Gastroenterol..

[7] Banerjee G., Ray A.K. (2017). The advancement of probiotics research and its application in fish farming industries. Res. Vet. Sci..

[8] Corr S.C., Hill C., Gahan C.G.M. (2009). Chapter 1 Understanding the Mechanisms by Which Probiotics Inhibit Gastrointestinal Pathogens. Advances in Food and Nutrition Research.

[9] Ghanei-Motlagh R., Mohammadian T., Gharibi D., Khosravi M., Mahmoudi E., Zarea M., El-Matbouli M., Menanteau-Ledouble S. (2020). Quorum quenching probiotics modulated digestive enzymes activity, growth performance, gut microflora, haemato-biochemical parameters and resistance against *Vibrio harveyi* in Asian seabass (*Lates calcarifer*). Aquaculture.

[10] Ghanei-Motlagh R., Mohammadian T., Gharibi D., Menanteau-Ledouble S., Mahmoudi E., Khosravi M., Zarea M., El-Matbouli M. (2019). Quorum Quenching Properties and Probiotic Potentials of Intestinal Associated Bacteria in Asian Sea Bass *Lates calcarifer*. Mar. Drugs.

[11] Lawrence C., Mason T. (2012). Zebrafish housing systems: A review of basic operating principles and considerations for design and functionality. ILAR J..

[12] Schreier H.J., Mirzoyan N., Saito K. (2010). Microbial diversity of biological filters in recirculating aquaculture systems. Curr. Opin. Biotechnol..

[13] Van Kessel M.A.H.J., Harhangi H.R., Flik G., Jetten M.S.M., Klaren P.H.M., den Camp H.J.M.O. (2011). Anammox bacteria in different compartments of recirculating aquaculture systems. Biochem. Soc. Trans..

[14] Kuypers M.M.M., Marchant H.K., Kartal B. (2018). The microbial nitrogen-cycling network. Nat. Rev. Microbiol..

[15] Masser M.P., Rakocy J., Losordo T.M. (1999). Recirculating Aquaculture Tank Production Systems Management of Recirculating Systems. https://cals.arizona.edu/azaqua/extension/Classroom/SRAC/453fs.pdf.

[16] Gonçalves A.A., Gagnon G.A. (2011). Ozone Application in Recirculating Aquaculture System: An Overview. Ozone Sci. Eng..

[17] Ramos M.A., Batista S., Pires M.A., Silva A.P., Pereira L.F., Saavedra M.J., Ozório R.O.A., Rema P. (2017). Dietary probiotic supplementation improves growth and the intestinal morphology of Nile tilapia. Animal.

[18] Kesselring J.C., Gruber C., Standen B., Wein S. (2019). Continuous and pulse-feeding application of multispecies probiotic bacteria in whiteleg shrimp, *Litopenaeus vannamei*. J. World Aquac. Soc..

[19] Standen B.T., Peggs D.L., Rawling M.D., Foey A., Davies S.J., Santos G.A., Merrifield D.L. (2016). Dietary administration of a commercial mixed-species probiotic improves growth performance and modulates the intestinal immunity of tilapia, *Oreochromis niloticus*. Fish. Shellfish Immunol..

[20] Krummenauer D., Poersch L., Romano L.A., Lara G.R., Encarnação P., Wasielesky W. (2014). The Effect of Probiotics in a *Litopenaeus vannamei* Biofloc Culture System Infected with *Vibrio parahaemolyticus*. J. Appl. Aquac..

[21] Herlemann D.P., Labrenz M., Jürgens K., Bertilsson S., Waniek J.J., Andersson A.F. (2011). Transitions in bacterial communities along the 2000 km salinity gradient of the Baltic Sea. ISME J..

[22] Rosén A. (1996). Denitrification by *Rhizobium meliloti*, Swedish University of Agricultural Sciences. https://www.osti.gov/etdeweb/servlets/purl/378189.

[23] Delmont T.O., Quince C., Shaiber A., Esen Ö.C., Lee S.T., Rappé M.S., McLellan S.L., Lücker S., Eren A.M. (2018). Nitrogen-fixing populations of Planctomycetes and Proteobacteria are abundant in surface ocean metagenomes. Nat. Microbiol..

[24] Sorokin D.Y., Lücker S., Vejmelkova D., Kostrikina N.A., Kleerebezem R., Rijpstra W.I.C., Damsté J.S.S., Le Paslier D., Muyzer G., Wagner M. (2012). Nitrification expanded: Discovery, physiology and genomics of a nitrite-oxidizing bacterium from the phylum *Chloroflexi*. ISME J..

[25] Hüpeden J., Wemheuer B., Indenbirken D., Schulz C., Spieck E. (2020). Taxonomic and functional profiling of nitrifying biofilms in freshwater, brackish and marine RAS biofilters. Aquac. Eng..

[26] Brailo M., Schreier H.J., McDonald R., Maršić-Lučić J., Gavrilović A., Pećarević M., Jug-Dujaković J. (2019). Bacterial community analysis of marine recirculating aquaculture system bioreactors for complete nitrogen removal established from a commercial inoculum. Aquaculture.

[27] Bartelme R.P., McLellan S.L., Newton R.J. (2017). Freshwater Recirculating Aquaculture System Operations Drive Biofilter Bacterial Community Shifts around a Stable Nitrifying Consortium of Ammonia-Oxidizing *Archaea* and Comammox *Nitrospira*. Front. Microbiol..

[28] Mohapatra S., Chakraborty T., Prusty A.K., Das P., Paniprasad K., Mohanta K.N. (2012). Use of different microbial probiotics in the diet of rohu, *Labeo rohita* fingerlings: Effects on growth, nutrient digestibility and retention, digestive enzyme activities and intestinal microflora. Aquac. Nutr..

